# Age May Not be a Barrier: Cementless Total Knee Arthroplasty Yields Equivalent or Superior PROMs in Older Adults at One Year

**DOI:** 10.1007/s43465-026-01883-2

**Published:** 2026-09-16

**Authors:** Benjamin E. Jevnikar, Khaled A. Elmenawi, Yuxuan Jin, Precious C. Oyem, Cleveland Clinic Adult Reconstruction Research Group, Nicolas S. Piuzzi

**Affiliations:** 1 Department of Orthopaedic Surgery, Cleveland Clinic, Cleveland, OH 44195 USA; 2 Cleveland Clinic Orthopaedic and Rheumatology Institute, 9500 Euclid Ave, A41Cleveland, Cleveland, OH 44195 USA

**Keywords:** Age, PROM, Total Knee Replacement, Total Knee Arthroplasty, Satisfaction

## Abstract

**Background:**

Cementless total knee arthroplasty (TKA) is increasingly being used across the age spectrum, yet its impact on patient-reported outcomes (PROMs) in older adults remains understudied. As value-based care initiatives prioritize PROMs such as pain relief, functional recovery, and satisfaction, understanding age-specific differences is critical.

**Methods:**

We analyzed 1772 patients who underwent unilateral, fully cementless primary TKA between 2016 and 2023 using a prospectively collected institutional registry. Patients were stratified by age (≤ 60, 60–70, > 70 years), and outcomes included KOOS subscales (Pain, Physical Function Shortform [PS], and Joint Replacement [JR]), minimal clinically important difference (MCID), Patient Acceptable Symptom State (PASS), and satisfaction at 1 year. Multivariable logistic regression adjusted for baseline PROMs and demographic covariates.

**Results:**

Older patients (> 70) had significantly higher baseline KOOS scores and were more likely to exhibit favorable PROM phenotypes compared to younger cohorts. At 1 year, all groups experienced substantial improvement, with the > 70 group achieving the highest absolute KOOS scores. Patients aged 60–70 had significantly lower odds of failing to meet MCID (KOOS JR: OR 0.58; p = 0.027) and PASS thresholds (KOOS Pain: OR 0.75; KOOS PS: OR 0.75) compared to those ≤ 60. Older adults demonstrated non-inferior performance across all metrics, including satisfaction, which exceeded 83% across all age groups (p = 0.88).

**Conclusion:**

Cementless TKA yields strong MCID and PASS threshold achievement and high satisfaction across all age groups. Patients over 70 demonstrated excellent outcomes, challenging traditional age-based concerns and may encourage the use of cementless fixation in appropriately selected older adults. However, long-term data evaluating implant survivorship remains warranted.

## Introduction

Cementless fixation in total knee arthroplasty (TKA) has seen renewed interest in recent years, driven by advances in implant design, porous coatings, and biologic fixation [1–3]. Although long considered the preferred fixation strategy for younger, high-demand patients with robust bone quality, cementless TKA is increasingly being offered to older adults as well, particularly in high-volume centers with favorable early results [1, 4, 5]. Registry data now reflect a steady rise in cementless utilization [5, 6], yet there remains ongoing debate regarding its suitability in older patients [4, 6, 7].

Historically, concerns regarding cementless TKA in older adults include the theoretical need for a longer biologic fixation period, potential challenges with early postoperative recovery, increased risk of intraoperative fracture, and questionable suitability in patients with osteoporosis or morbid obesity who may require rapid postoperative mobilization [8, 9]. However, most studies on cementless fixation focus on implant survivorship and radiographic endpoints, with relatively limited evaluation of patient-reported outcome measures (PROMs) across different age strata [7, 8]. As value-based care metrics increasingly emphasize functional recovery, pain relief, and patient satisfaction, a better understanding of how age influences PROMs after cementless TKA is essential and can ultimately help guide shared decision-making and patient counseling.

Although an increasing number of studies have compared perioperative complications, implant survivorship, and healthcare utilization between cemented and cementless primary TKA[10, 11], there remains a need for a more detailed assessment of clinically meaningful differences in PROMs, especially given the growing emphasis on value-based reimbursement and the widespread adoption of PROMs to quantify postoperative improvement. Accordingly, the purpose of this study was to evaluate patient-reported outcomes at one year following cementless TKA, stratified by age. Specifically, we aimed to: (1) compare baseline and one-year KOOS scores across age groups; (2) assess differences in the likelihood of achieving MCID and PASS thresholds; and (3) determine whether age was associated with patient satisfaction following cementless TKA.

## Methods

### Study Design and Patient Population

**Fig. 1:**
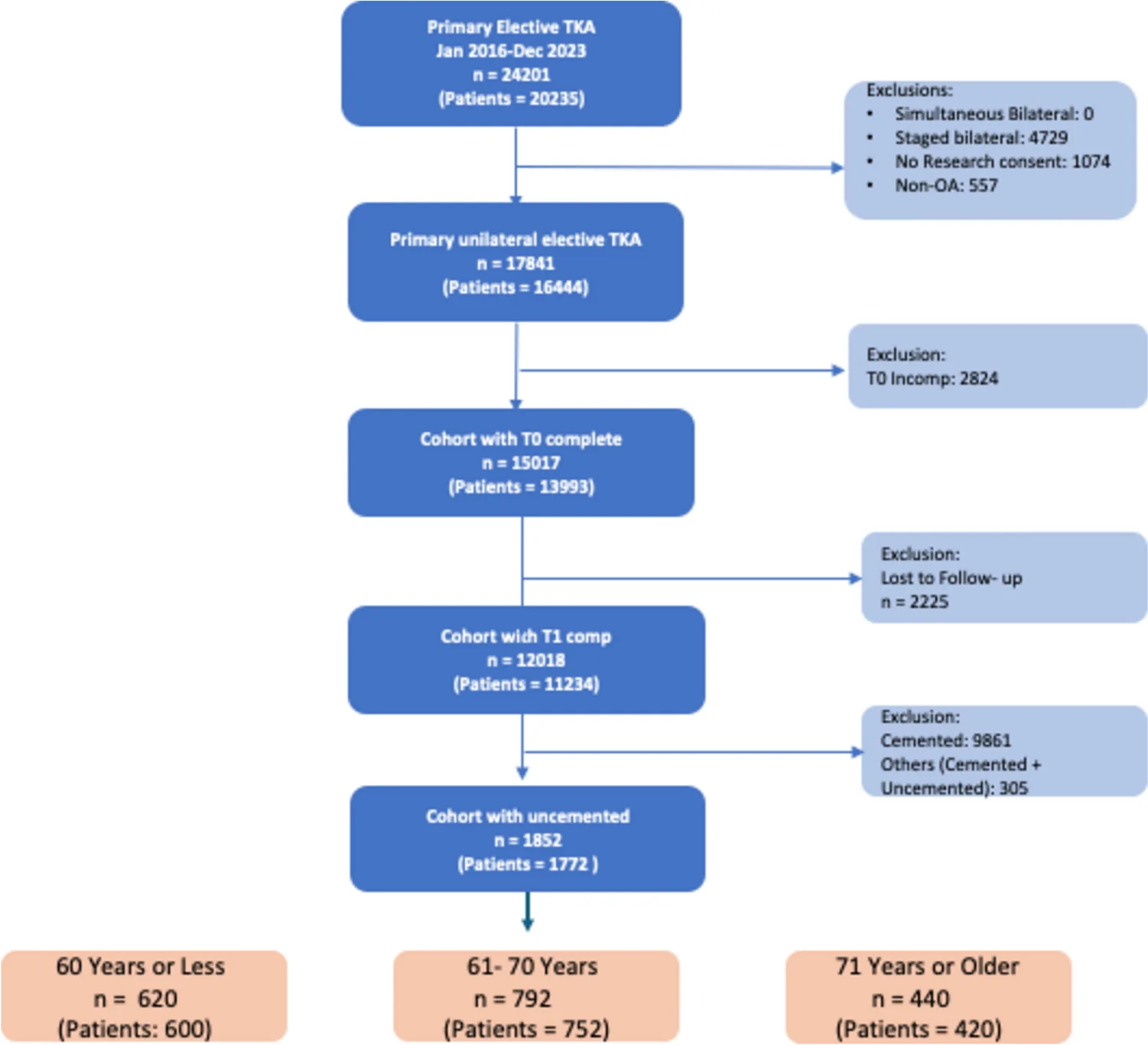
Strengthening the Reporting of Observational Studies in Epidemiology (STROBE) diagram

Using a validated, prospectively collected institutional registry [12–14], we analyzed a cohort of patients who underwent unilateral, fully cementless primary TKA for osteoarthritis at a single academic health system between 2016 and 2023. The decision to use cementless fixation was based on surgeon preference, which generally incorporated a combination of intraoperative assessment of bone quality, preoperative radiographic bone density, patient age, and anticipated activity level rather than a single predetermined criterion. All patients were enrolled in an institutional arthroplasty registry and completed PROMs preoperatively and at 1-year follow-up. Patients were excluded if they underwent bilateral TKA, refused to participate in research, or if baseline PROM data were unavailable (Fig. 1).

Patients were stratified into three groups based on age at the time of surgery: ≤ 60 years (n = 600), 60–70 years (n = 752), and > 70 years (n = 420) (Table 1**; **Fig. 1). Demographic variables included age, sex, body mass index (BMI), race, education, smoking status, area deprivation index (ADI) [15–21], Charlson Comorbidity Index (CCI), insurance, anesthesia type, and Narx score [22–24]. Baseline PROMs were assessed to evaluate preoperative symptom burden across age groups.

**Table 1:** Baseline demographic characteristics of patient cohort

Variable	Level	Total (N = 1772)	< = 60 (N = 600)	(60,70] (N = 752)	> 70 (N = 420)	P-value
Age		64.0 [58.0;70.0]	56.0 [52.0;58.0]	65.0 [63.0;68.0]	74.0 [72.0;78.0]	0.000
Sex	Male	1002 (56.5%)	295 (49.2%)	458 (60.9%)	249 (59.3%)	< 0.001
	Female	770 (43.5%)	305 (50.8%)	294 (39.1%)	171 (40.7%)	
BMI		31.2 [27.5;35.9]	33.0 [28.6;37.7]	31.2 [27.9;35.7]	29.2 [26.3;32.8]	< 0.001
Race	White	1546 (89.7%)	491 (85.1%)	674 (91.8%)	381 (92.5%)	< 0.001
	Black	37 (2.15%)	13 (2.25%)	13 (1.77%)	11 (2.67%)	
	Other	140 (8.13%)	73 (12.7%)	47 (6.40%)	20 (4.85%)	
Education		14.0 [12.0;16.0]	14.0 [12.0;16.0]	14.0 [12.0;16.0]	15.0 [12.0;16.0]	0.044
Smoking	Never	1039 (58.6%)	368 (61.3%)	448 (59.6%)	223 (53.1%)	< 0.001
	Quit 6 m +	584 (33.0%)	154 (25.7%)	253 (33.6%)	177 (42.1%)	
	Quit < 6 m	47 (2.65%)	23 (3.83%)	13 (1.73%)	11 (2.62%)	
	Current	102 (5.76%)	55 (9.17%)	38 (5.05%)	9 (2.14%)	
ADI		44.0 [28.0;61.0]	48.0 [31.0;67.0]	43.0 [27.0;60.0]	40.0 [24.8;55.0]	< 0.001
CCI		0.00 [0.00;2.00]	0.00 [0.00;1.00]	0.00 [0.00;2.00]	1.00 [0.00;2.00]	< 0.001
Insurance	Private	845 (50.3%)	467 (81.9%)	353 (48.6%)	25 (6.53%)	
	Medicaid	77 (4.59%)	57 (10.0%)	20 (2.75%)	0 (0.00%)	
	Medicare	736 (43.8%)	33 (5.79%)	347 (47.8%)	356 (93.0%)	
	Other	21 (1.25%)	13 (2.28%)	6 (0.83%)	2 (0.52%)	
Anesthesia	General	319 (18.7%)	93 (15.8%)	139 (19.0%)	87 (22.7%)	0.008
	Spinal	1316 (77.1%)	480 (81.5%)	555 (75.7%)	281 (73.2%)	
	Other	71 (4.16%)	16 (2.72%)	39 (5.32%)	16 (4.17%)	
NARX		110 [0.00;190]	110 [0.00;190]	110 [0.00;190]	110 [0.00;190]	0.371

Data reported as median [IQR] or n (%). *BMI* body mass index; *ADI* area deprivation index; *CCI* Charlson comorbidity index; *NARX* narcotic opioid risk score

### Outcome Measures

The primary outcomes were one-year postoperative PROMs and achievement of clinically meaningful thresholds. Specifically, we assessed the minimal clinically important difference (MCID) and the Patient Acceptable Symptom State (PASS) for three KOOS subscales: KOOS Pain, KOOS Physical Function Shortform (KOOS PS), and KOOS Joint Replacement (KOOS JR). The VR-12 Mental Component Score (MCS) was also analyzed at baseline. Binary patient satisfaction at one year was assessed using a validated PASS-derived anchor question [25–27].

PROM phenotype categories were used to summarize baseline symptom burden [27–29]. Patients were classified into eight mutually exclusive categories based on whether they met positive thresholds for pain (KOOS Pain ≥ 70), function (KOOS PS ≥ 65), and mental health (VR-12 MCS ≥ 50). These categories were used descriptively to compare PROM clustering across age groups.

### Statistical Analysis

Patients with missing baseline or one-year PROM data were excluded from the relevant analyses, resulting in a complete-case analytic approach. Descriptive statistics were used to compare demographic and baseline PROM characteristics across the three age groups using chi-square and ANOVA or Kruskal–Wallis tests, as appropriate. Change in PROMs from baseline to 1 year was analyzed using within-group comparisons and summarized using medians with interquartile ranges. Multivariable logistic regression was used to evaluate the association between age group and the risk of failing to achieve MCID or PASS thresholds. Satisfaction at 1 year was also modeled using logistic regression. All models were adjusted for potential confounders, including sex, race, insurance type, CCI, and baseline PROM scores. The ≤ 60-year group served as the referent in all models. Odds ratios (ORs) with 95% confidence intervals (CIs) were reported. A p-value < 0.05 was considered statistically significant. All analyses were conducted using R version 4.2.3 (R Foundation for Statistical Computing, Vienna, Austria).

## Results

### PROMs at Baseline vs. 1-Year

At baseline, median KOOS Pain was highest in the > 70 group at 45.8 [36.1–55.6], compared to 41.7 [33.3–52.8] in the 60–70 group and 38.9 [30.6–50.0] in the ≤ 60 group (p < 0.001). Similarly, KOOS JR was highest among patients > 70 (47.5 [39.6–57.1]) and lowest in the ≤ 60 group (44.9 [36.9–52.5]; p < 0.001). KOOS PS was slightly higher in the 60–70 and > 70 groups (53.9 [45.6–61.4]) than in the ≤ 60 group (51.5 [42.1–59.7]; p = 0.007). This pattern was further supported by PROM phenotype distributions. The least favorable phenotype (Pain-, PS-, MCS-) was most common in younger patients (25.2% in ≤ 60 vs 19.8% in > 70). In contrast, the most favorable phenotype (Pain + , PS + , MCS +) was disproportionately more prevalent in older adults: 32.6% in patients > 70, compared to only 23.2% in those ≤ 60 (p < 0.001) (Table 2).

**Table 2:** Baseline and one year (1y) knee injury and osteoarthritis outcome score (KOOS) scores

Variable	Level	Total (N = 1772)	< = 60 (N = 600)	(60,70] (N = 752)	> 70 (N = 420)	P-value
Baseline KOOS Pain		41.7 [33.3;52.8]	38.9 [30.6;50.0]	41.7 [33.3;52.8]	45.8 [36.1;55.6]	< 0.001
Baseline KOOS PS		53.9 [45.6;59.7]	51.5 [42.1;59.7]	53.9 [45.6;61.4]	53.9 [45.6;61.4]	0.007
Baseline KOOS JR		47.5 [39.6;54.8]	44.9 [36.9;52.5]	47.5 [39.6;54.8]	47.5 [39.6;57.1]	< 0.001
1y KOOS Pain		88.9 [72.2;97.2]	83.3 [69.4;94.4]	88.9 [75.0;97.2]	88.9 [77.8;97.2]	< 0.001
1y KOOS PS		75.1 [66.4;85.2]	72.5 [64.7;85.2]	78.0 [68.2;85.2]	78.0 [66.4;89.5]	< 0.001
1y KOOS JR		76.3 [68.3;84.6]	73.3 [63.8;84.6]	76.3 [68.3;92.0]	79.9 [68.3;92.0]	< 0.001
PROM Phenotype	Pain + PS + MCS +	501 (28.3%)	139 (23.2%)	225 (29.9%)	137 (32.6%)	< 0.001
	Pain + PS + MCS-	250 (14.1%)	72 (12.0%)	109 (14.5%)	69 (16.4%)	
	Pain + PS- MCS +	54 (3.05%)	13 (2.17%)	18 (2.39%)	23 (5.48%)	
	Pain + PS- MCS-	40 (2.26%)	8 (1.33%)	20 (2.66%)	12 (2.86%)	
	Pain- PS + MCS +	177 (9.99%)	73 (12.2%)	79 (10.5%)	25 (5.95%)	
	Pain- PS + MCS-	138 (7.79%)	56 (9.33%)	62 (8.24%)	20 (4.76%)	
	Pain- PS- MCS +	248 (14.0%)	88 (14.7%)	109 (14.5%)	51 (12.1%)	
	Pain- PS- MCS-	364 (20.5%)	151 (25.2%)	130 (17.3%)	83 (19.8%)	
PASS Question	No	281 (16.2%)	105 (17.7%)	113 (15.3%)	63 (15.5%)	0.450
	Yes	1458 (83.8%)	488 (82.3%)	626 (84.7%)	344 (84.5%)	

*PROM* patient-reported outcome measure; *PS* Physical Function Shortform; *JR* Joint replacement; *MCS* Mental Component Score; *PASS* Patient-acceptable symptom state

By one year, all age groups experienced substantial improvements in KOOS scores. KOOS Pain improved to 83.3 [69.4–94.4] in the ≤ 60 group, 88.9 [75.0–97.2] in the 60–70 group, and 88.9 [77.8–97.2] in the > 70 group (p < 0.001). KOOS PS also improved across age groups, reaching 72.5 [64.7–85.2] in patients ≤ 60, 78.0 [68.2–85.2] in those 60–70, and 78.0 [66.4–89.5] in those > 70 (p < 0.001). KOOS JR at one year was lowest in the ≤ 60 group (73.3 [63.8–84.6]) and highest in the > 70 group (79.9 [68.3–92.0]; p < 0.001).

### MCID and PASS Threshold Achievement

**Fig. 2:**
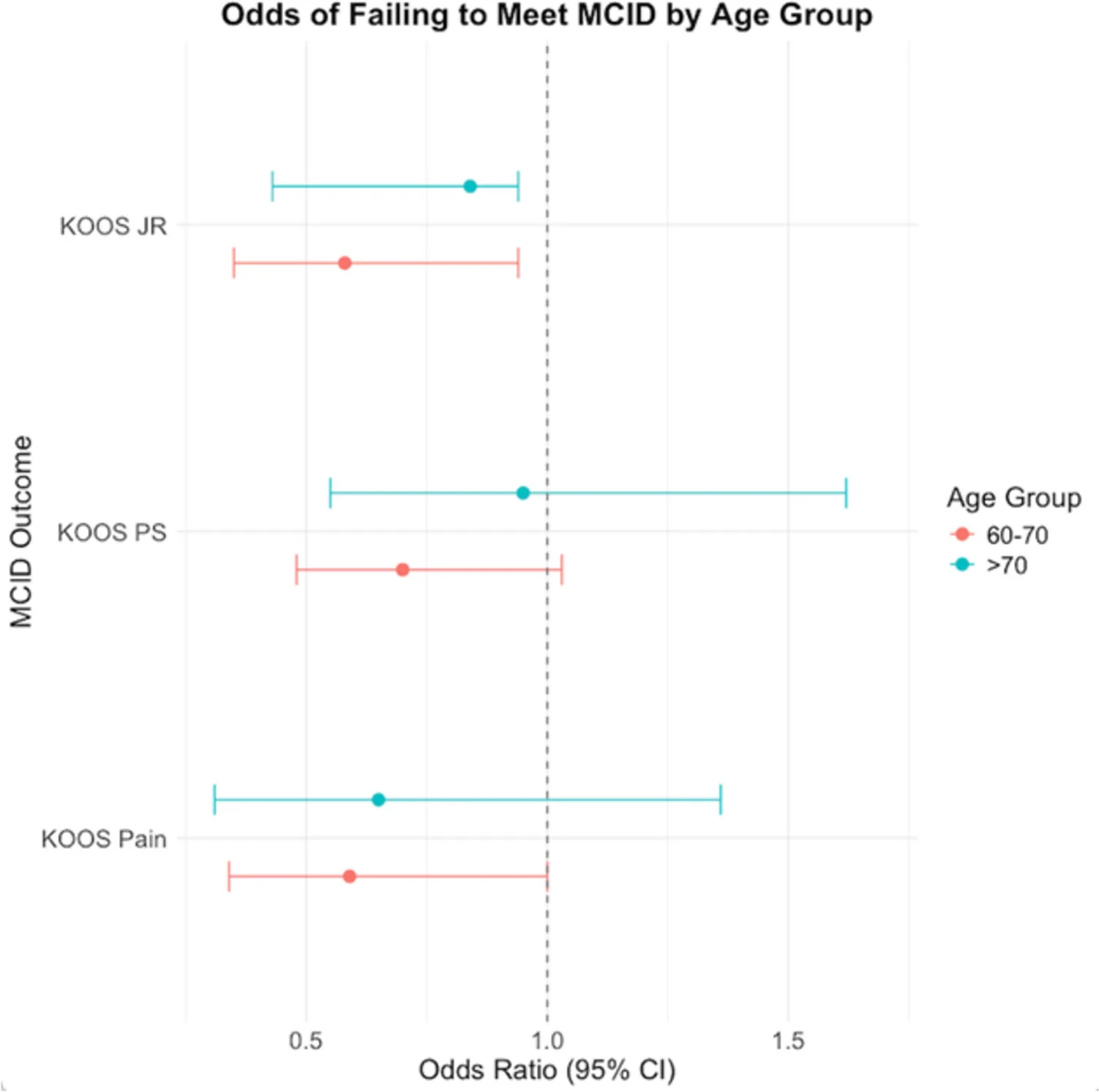
Odds of failing to achieve the minimal clinically important difference (MCID) (age group versus <  = 60 group. KOOS: knee injury and osteoarthritis outcome score. PS: Physical Function Shortform. JR: Joint replacement. MCS: Mental Component Score

Compared to patients > 60, those in the 60–70 group had significantly lower odds of failing to achieve the MCID for KOOS JR (OR 0.58, 95% CI 0.35–0.94; *p* = 0.027). No statistically significant differences in MCID failure rates were observed between the > 70 and ≤ 60 age groups across KOOS Pain (p = 0.252), KOOS PS (p = 0.841), or KOOS JR (p = 0.599) (Fig. 2).

**Fig. 3:**
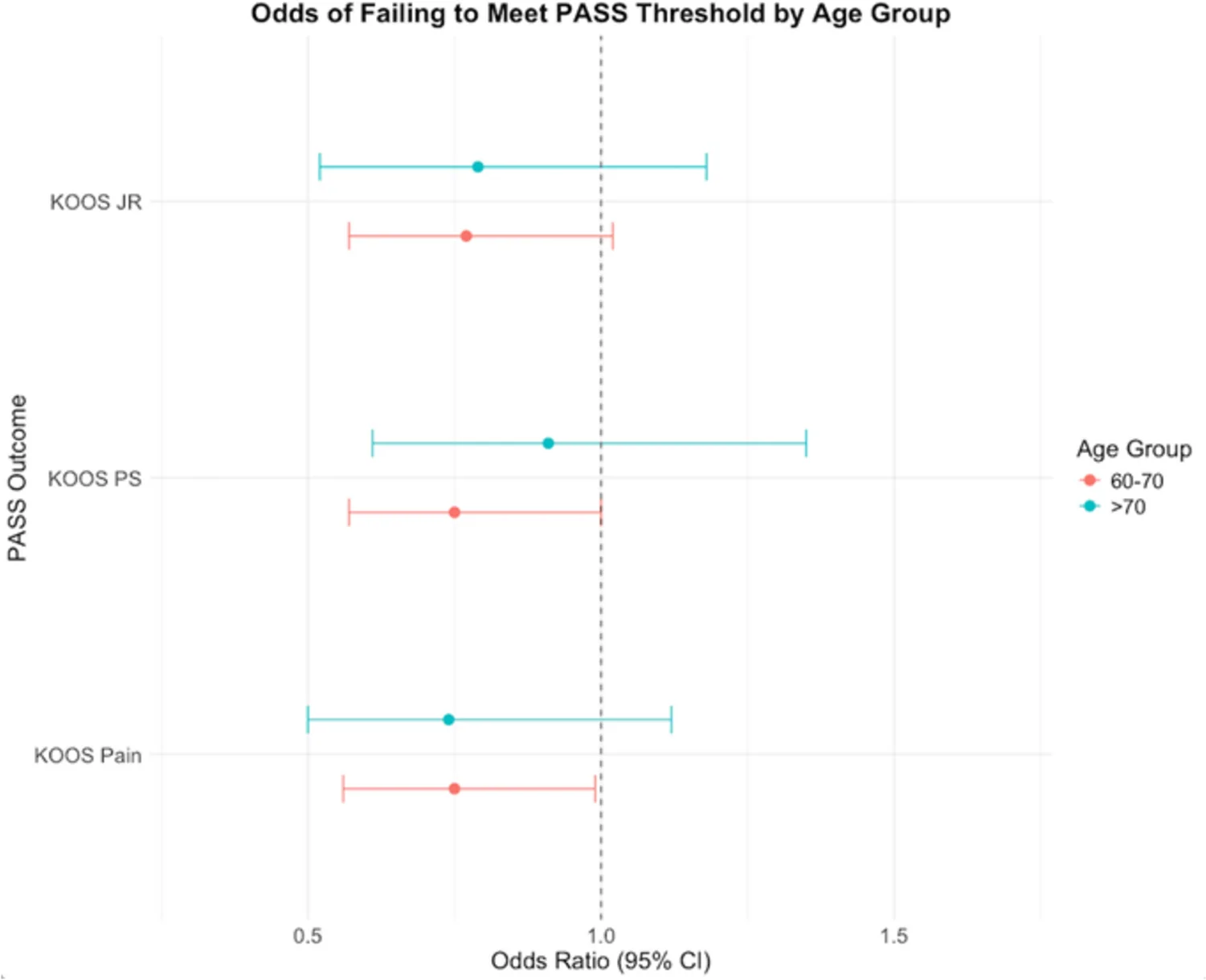
Odds of failing to achieve the patient-acceptable symptom state (PASS) threshold (age group versus <  = 60 group. KOOS: knee injury and osteoarthritis outcome score. PS: Physical Function Shortform. JR: Joint replacement. MCS: Mental Component Score

Patients aged 60–70 were significantly less likely to fail the PASS threshold for KOOS Pain (OR 0.75, 95% CI 0.56–0.99; *p* = 0.042) and KOOS PS (OR 0.75, 95% CI 0.57–1.00; *p* = 0.046), but no difference was observed for KOOS JR (p = 0.069). There were no statistically significant differences between patients > 70 and those ≤ 60 in PASS threshold achievement for any KOOS domain (*p* > 0.15 for all) (Fig. 3).

### Satisfaction

**Fig. 4:**
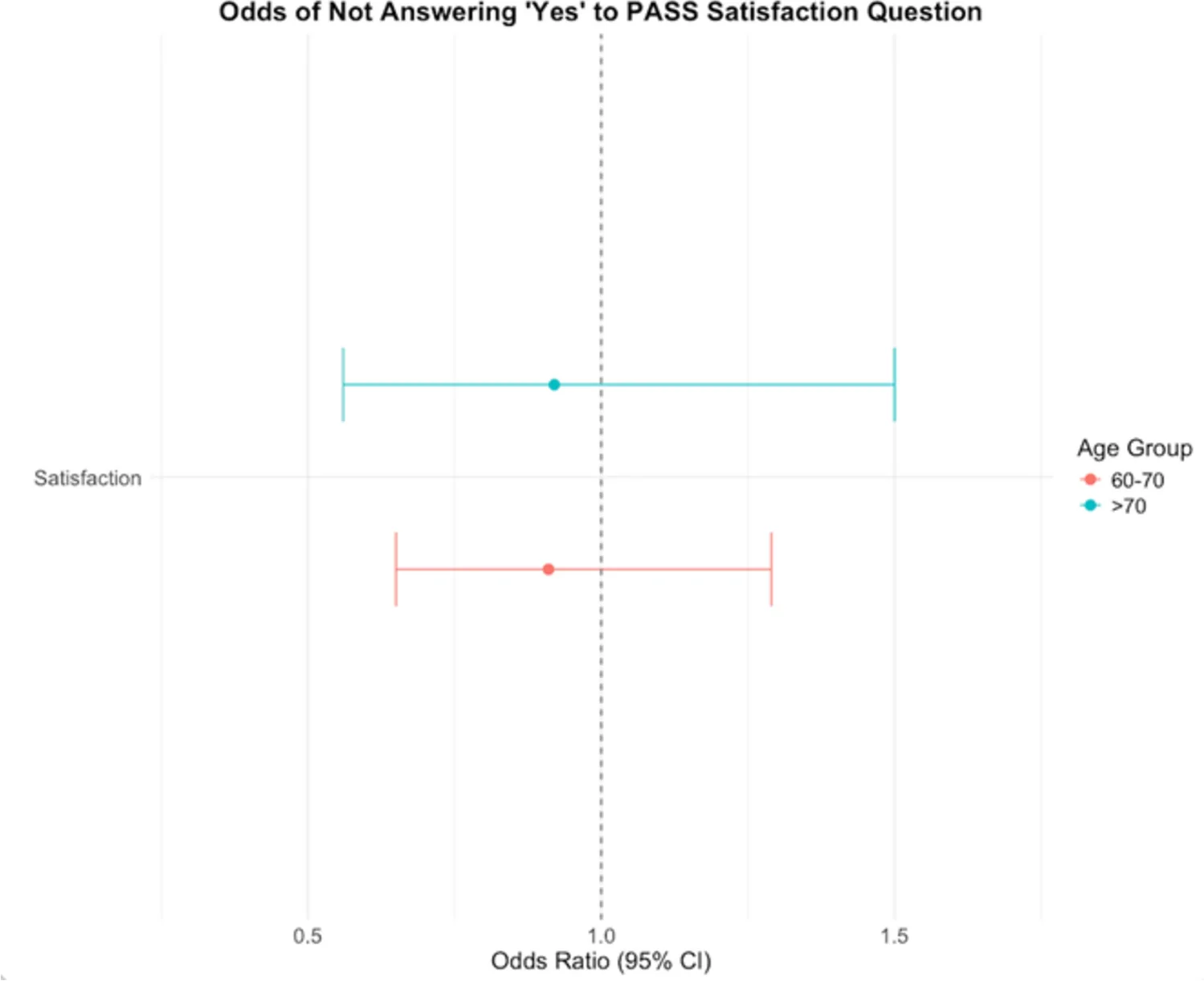
Odds of failing to answer ‘Yes’ in the patient-acceptable symptom state (PASS) questionnaire, indicating dissatisfaction

One-year satisfaction, based on a binary PASS anchor question, was high and consistent across all age groups (overall: 83.8%; ≤ 60: 82.3%; 60–70: 84.7%; > 70: 84.5%). Age group was not associated with satisfaction in adjusted models (60–70 vs ≤ 60: OR 0.91, 95% CI 0.65–1.29; > 70 vs ≤ 60: OR 0.92, 95% CI 0.56–1.50; overall* p* = 0.88) (Fig. 4).

## Discussion

With advances in implant design, porous coating, and biologic fixation, cementless TKA has gained significant traction in recent years [1–3]. Yet, the impact of cementless TKA on PROMs across the age spectrum has not been adequately described. In an era of value-based care, this deficiency is particularly problematic. In this large cohort of patients undergoing cementless TKA, patients aged 60–70 were less likely to fail the MCID for KOOS JR and the PASS threshold for KOOS Pain and PS. However, each age group had comparable odds of achieving clinically meaningful improvements in all other PROMs, suggesting age alone has minimal impact on PROMs following cementless TKA.

Our findings align with and expand upon a growing body of literature demonstrating favorable outcomes of cementless TKA in older adults. Prior studies have shown that patients ≥ 75 years can achieve clinical outcomes equivalent to younger patients in terms of pain relief, functional recovery, and quality of life, with no increased risk of aseptic loosening or implant failure at short- to mid-term follow-up [7, 8, 10, 30]. Goh et al. [8] and Maniar et al. [7] specifically reported no age-related decrement in clinical outcome scores or survivorship among patients over 70 undergoing cementless TKA. However, both studies were limited by smaller sample sizes (n = 480 and 347, respectively) and did not include age-stratified multivariable modeling within a fully cementless cohort. Our study analyzed a substantially larger cohort of 1772 cementless TKAs and evaluated MCID and PASS achievement across age groups, adjusting for relevant confounders. We found that patients > 70 not only experienced excellent one-year KOOS scores, but in fact outperformed younger cohorts across pain, function, and joint replacement domains. These findings provide strong evidence that cementless fixation can be safely and effectively utilized in appropriately selected older patients, challenging traditional concerns about age-related limitations and supporting its broader adoption in this population.

One of the more unexpected findings was that older adults began with better baseline KOOS Pain and PS scores compared to younger patients. This contrasts with traditional assumptions that older patients present with more advanced disease or diminished function. However, several studies have reported similar trends, noting that younger TKA candidates, particularly those under 60, often have lower preoperative PROMs and mental health scores despite similar radiographic severity [31–33]. This has been attributed to heightened symptom sensitivity, increased functional demand in younger individuals, or differences in decision making for surgical indications or PROM thresholds. Our PROM phenotype data further support this interpretation, showing that younger patients were more likely to cluster in the least favorable symptom group (Pain-, PS-, MCS-), while older adults disproportionately met thresholds for favorable PROM phenotypes. These differences in baseline status may help explain why younger patients, despite greater absolute improvements, still reported lower absolute KOOS scores at one year. However, this registry-based finding may also reflect selection bias, as surgeons may defer arthroplasty in younger patients until symptom burden is substantial, whereas older patients may proceed to surgery at a relatively earlier symptomatic threshold.

Achievement of MCID and PASS thresholds provides important context to raw PROM scores. Patients aged 60–70 had significantly lower odds of failing to achieve MCID for KOOS JR compared to those ≤ 60, while patients > 70 demonstrated non-inferior performance across all domains. Similar patterns were observed for PASS achievement, with patients in the 60–70 group having significantly lower odds of PASS threshold failure compared to the ≤ 60 cohort. These findings echo prior evidence suggesting that age alone is not a major determinant of poor outcome and that patient selection based on physiologic rather than chronologic age may be more appropriate [34, 35]. Moreover, our use of multivariable modeling to isolate the impact of age adds a layer of rigor that many prior studies have lacked, further supporting the clinical validity of cementless fixation in older adults.

From a clinical and policy standpoint, these findings are highly relevant. Satisfaction rates were uniformly high across age groups, and age was not an independent predictor of dissatisfaction. Additionally, while the substantial clinical benefit (SCB) was not calculated for KOOS-JR, our findings of non-inferior MCID and PASS threshold achievement for KOOS-JR provide important context for the new CMS Patient-Reported Outcomes Performance-Based Measures (PRO-PMs) policy. As cementless fixation becomes more widespread, registry-based PROMs will increasingly influence policy, reimbursement, and performance metrics. These data can help inform shared decision-making, surgeon counseling, and payer considerations, reinforcing that well-selected older patients remain excellent candidates for cementless fixation.

This study is not without limitations. First, this study did not include a contemporaneous cemented TKA comparison group. Accordingly, our findings should be interpreted as age-stratified outcomes within a fully cementless cohort rather than as a direct comparison between fixation strategies. However, this design allowed us to isolate the effect of age on patient-reported outcomes following cementless fixation without the confounding influence of mixed implant constructs. As with any registry-based analysis, selection bias may influence which patients were chosen for cementless fixation. We excluded patients lacking baseline PROMs, which may also introduce bias. While follow-up was limited to one year, numerous literature supports the idea that one-year PROMs are as clinically reliable and meaningful as longer term PROMs [36–38]. Additionally, the decision to opt for cementless TKA was based on surgeons’ preference, which is often influenced by bone quality and patient activity level, which may bias our findings. While this reflects real-world clinical decision-making, it may limit generalizability to patients with severe osteoporosis or poor bone stock. Furthermore, although we adjusted for numerous confounders, residual confounding may persist. Finally, this analysis focused on patient-reported outcomes and did not specifically evaluate perioperative complications, reoperations, or implant survivorship. Although prior registry and institutional studies have demonstrated comparable short- to mid-term complication rates between cementless and cemented TKA [10, 11], complication profiles were beyond the scope of the present investigation. Future studies integrating PROMs with complication and survivorship endpoints will be important to further contextualize these findings. Nonetheless, the large cohort size, prospective PROM collection, and detailed multivariable analyses provide a strong foundation for these conclusions.

## Conclusion

In this large, cohort-based analysis of cementless TKA, we found that older adults achieved functional outcomes, satisfaction, and clinically meaningful improvement comparable to those of younger patients. Patients over 70 demonstrated the highest absolute KOOS scores at one year, were more likely to exhibit favorable baseline PROM phenotypes, and showed non-inferior rates of achieving MCID and PASS thresholds after adjustment for clinical and demographic factors. These findings may further support increased adoption of cementless TKA in older individuals. However, long-term survivorship data are still warranted. As cementless utilization continues to expand, our results provide high-quality evidence to inform patient selection, counseling, and value-based surgical decision-making across the age spectrum.
